# Supramammillary serotonin reduction alters place learning and concomitant hippocampal, septal, and supramammillar theta activity in a Morris water maze

**DOI:** 10.3389/fphar.2015.00250

**Published:** 2015-10-29

**Authors:** J. Jesús Hernández-Pérez, Blanca E. Gutiérrez-Guzmán, Miguel Á. López-Vázquez, María E. Olvera-Cortés

**Affiliations:** ^1^Laboratorio de Neurofisiología Experimental, División de Neurociencias, Centro de Investigación Biomédica de Michoacán, Instituto Mexicano del Seguro SocialMorelia, México; ^2^Laboratorio de Neuroplasticidad de los Procesos Cognitivos, División de Neurociencias, Centro de Investigación Biomédica de Michoacán, Instituto Mexicano del Seguro SocialMorelia, México; ^3^Instituto de Física y Matemáticas, Universidad Michoacana de San Nicolás de HidalgoMorelia, México

**Keywords:** supramammillary nucleus, serotonin, septum, hippocampus, theta activity, spatial learning

## Abstract

Hippocampal theta activity is related to spatial information processing, and high-frequency theta activity, in particular, has been linked to efficient spatial memory performance. Theta activity is regulated by the synchronizing ascending system (SAS), which includes mesencephalic and diencephalic relays. The supramamillary nucleus (SUMn) is located between the *reticularis pontis oralis* and the medial septum (MS), in close relation with the posterior hypothalamic nucleus (PHn), all of which are part of this ascending system. It has been proposed that the SUMn plays a role in the modulation of hippocampal theta-frequency; this could occur through direct connections between the SUMn and the hippocampus or through the influence of the SUMn on the MS. Serotonergic raphe neurons prominently innervate the hippocampus and several components of the SAS, including the SUMn. Serotonin desynchronizes hippocampal theta activity, and it has been proposed that serotonin may regulate learning through the modulation of hippocampal synchrony. In agreement with this hypothesis, serotonin depletion in the SUMn/PHn results in deficient spatial learning and alterations in CA1 theta activity-related learning in a Morris water maze. Because it has been reported that SUMn inactivation with lidocaine impairs the consolidation of reference memory, we asked whether changes in hippocampal theta activity related to learning would occur through serotonin depletion in the SUMn, together with deficiencies in memory. We infused 5,7-DHT bilaterally into the SUMn in rats and evaluated place learning in the standard Morris water maze task. Hippocampal (CA1 and dentate gyrus), septal and SUMn EEG were recorded during training of the test. The EEG power in each region and the coherence between the different regions were evaluated. Serotonin depletion in the SUMn induced deficient spatial learning and altered the expression of hippocampal high-frequency theta activity. These results provide evidence in support of a role for serotonin as a modulator of hippocampal learning, acting through changes in the synchronicity evoked in several relays of the SAS.

## Introduction

Hippocampal theta activity has been related to processing of spatial information in different behavioral paradigms in various animal species (Ammassari-Teule et al., 1991; McNaughton et al., 2006) as well as in human beings (Klimesch et al., 1994; Klimesch, 1999; Caplan et al., 2001; Ekstrom et al., 2005; Lega et al., 2014). The relation of theta activity and place learning has been also studied; changes in power and/or frequency of the hippocampal theta activity have been associated with efficient learning during place learning tests in the Morris maze (Pan and McNaughton, 1997; Olvera-Cortes et al., 2002, 2004; Olvera-Cortés et al., 2012; Buzsaki, 2005; Ruan et al., 2011), conditioning (Berry and Seager, 2001; Berry and Hoffmann, 2011), working memory (Mitchell et al., 1982), and novelty detection (Aggleton and Brown, 1999; Vinogradova, 2001), among others. Moreover, deficient spatial memory has been observed after the reductions in the frequency of hippocampal theta activity (Winson, 1978; Pan and McNaughton, 1997).

Theta activity is modulated by a group of mesencephalic-diencephalic structures called the synchronizing ascending system (SAS) (Bland et al., 1990; Kirk et al., 1996; Leranth et al., 1999; Woodnorth et al., 2003). Theta activity can be generated in the hippocampus by stimulation of the nucleus *reticularis pontis oralis* (RPOn) both in anesthetized and in awake animals (Vertes, 1982, 1986). It was proposed that the RPOn theta modulation spreads through the tegmental pedunculopontine nucleus (TPPn) to the hypothalamic relays, the supramammillary (SUMn) and posterior hypothalamic (PHn) nuclei (Takano and Hanada, 2009). Because of to the tonic firing of RPOn neurons, the rhythmical firing of SUMn cells, and the result from inactivating SUMn, it was proposed that SUMn convert the tonic input received from the RPOn into a rhythmical pattern, which is relayed to the medial septum (MS), considered the pacemarker of the theta activity (Gogolak et al., 1968; Petsche et al., 1968; Andersen et al., 1979; Kirk and McNaughton, 1991; Kirk and Mackay, 2003). In support of this hypothesis, procaine infusions into (medial) SUMn induce a decrease in the frequency of hippocampal theta activity elicited by stimulation of RPOn in awake or in anesthetized rats (Kirk and McNaughton, 1993; McNaughton et al., 1995). Moreover, the rhythmic activity in the SUMn elicited by infusing carbacol into the RPOn persists after either the infusion of procaine into the MS or the bilateral transection of the communication pathways between SUMn and the MS (Kirk et al., 1996; Kirk, 1997). Additionally, an efferent influence from MS, which induce the deceleration of theta frequency-related firing in SUMn neurons, was observed (Kocsis, 2006; Kocsis and Kaminski, 2006); this influence could originate in the reciprocal connections between the two nuclei (Vertes, 1992), possibly through a GABAergic, input from the lateral septum (LS) on the (lateral) SUMn (Leranth and Kiss, 1996).

The SUMn has been related to information processing in memory. SUMn c-fos activity increases in spatial tasks (exploration, reference memory, and working memory) in the Morris water maze (Santin et al., 2003). Additionally, SUMn inactivation through the micro infusion of TTX induces deficiencies in reference memory retrieval (when TTX is applied in the seventh day of training, but not in the fourth day of training) and deficiencies in spatial working memory (Aranda et al., 2008). Furthermore, inactivation of SUMn with lidocaine impairs memory retrieval and consolidation in spatial memory tasks (Shahidi et al., 2004a). These results remarkably suggest that the SUMn functions in spatial information processing, although a relationship between SUMn and spatial learning is less clear (Santin et al., 2003). One study explored the relation between the SUMn, hippocampal theta activity and learning. Infusion of cholrdiazepoxide (CDP) into the (medial) SUMn had modest effects on theta activity and place learning in Morris water maze (Pan and McNaughton, 1997). However, after lidocaine inactivation of MS and the concomitant lack of hippocampal theta activity, both place learning and the rhythmicity of hippocampal theta activity (7.7 Hz) were restored by using the SUMn oscillation to rhythmically stimulate the fornix (McNaughton et al., 2006). This study demonstrated the relevance of both the SUMn and theta activity for place learning.

Similarly to the other relay nuclei of the SAS and the hippocampus, the SUM receives serotonergic axons both from medial and dorsal raphe nuclei (Vertes, 1988, 1992). The role of the serotonin originated in the raphe nuclei in desynchronizing of the hippocampal EEG is well documented. Briefly, stimulation of the medial raphe nucleus (MRn) desynchronizes the hippocampal EEG through the action of serotonin, whereas the electrolytic lesions of the same nucleus induce hippocampal EEG with a higher magnitude and longer duration, which is also present during immobility, in rats (Assaf and Miller, 1978; Maru et al., 1979). Furthermore, mucimol, buspirone and 8-hydroxy-2-(di-*n*-propyl-amino)-tetralin (8-OH-DPAT), a 5-HT1_A_ agonist, injections in MRn, induce persistent theta activity in the hippocampus of anesthetized rats, through the inhibition of serotonergic neurons (Vertes et al., 1994; Kinney et al., 1995). Thus, the serotonin can act on the SAS through many relays, or directly on the hippocampus to regulate theta activity; as a negative regulator of theta rhythmicity, serotonin could contribute to the fine-tuning of theta activity in the SUMn and thus influence on the upper relays, principally the MS and the hippocampus.

The role of serotonin as a modulator of learning has been extensively studied, although a complex picture emerges from the various papers possibly due to differences in learning tasks as well as differences in experimental strategies to manipulate the cerebral or regional serotonin activity, because of these factors, impairment, no effect or improvement in learning tasks has been reported after serotonin manipulations. Impairment in water maze tests was observed both after intra-septal or intra-hippocampal infusions of 8-OH-DPAT (Carli et al., 1992; Carli and Samanin, 1992; Bertrand et al., 2000). It has also been reported that intra-septal infusion of 8-OH-DPAT causes deficient spatial working memory (Jeltsch et al., 2004). In contrast, improvement in working memory and conditioning as well as improvement in place learning has been reported after reductions in cerebral, prefrontal and hippocampal serotonin (Altman et al., 1989; Pérez-Vega et al., 2000; Sarihi et al., 2000; Gutiérrez-Guzman et al., 2011). Additionally, a relation between the serotonergic modulation of theta and hippocampal-dependent place learning has been found (Gutiérrez-Guzman et al., 2011; Lopez-Vazquez et al., 2014). Moreover, reduction of serotonin content in the SUMn/PHn induced place learning deficiencies associated with a lack of learning-related increases in high-frequency hippocampal theta activity through the training (Gutiérrez-Guzman et al., 2012). Thus, the SUMn is a relay of the SAS participating in the modulation of hippocampal theta activity, and it is at least partially involved in place learning consolidation and/or recovery; it also receive serotonergic inputs, which could modulate the fine-tuning of hippocampal theta activity. However, despite the above, the effects of serotonin SUMn depletion alone on both spatial learning and on the characteristics of hippocampal, septal and SUMn theta activity during place learning have not been evaluated. The aim of the present work was to evaluate the consequences of serotonin depletion in the SUMn on place learning and the concomitant theta activity recorded from the SUMn, medial septum (MS), dentate gyrus (DG), and CA1, during the training in the Morris maze, in the rat.

## Methods

### Animals

Seventeen male, 4-months-old Sprague Dawley rats were used. The rats were maintained under standard facility conditions, and all of the experiments were conducted in accordance with the National Institutes of Health Guide for the Care and Use of Laboratory Animals (NIH Publication No. 80-23) and for the “Norma Oficiál Mexicana” for the use of experimental animals (NOM-062-ZOO-1999). All of the experiments were and approved by the Research Ethics Committee of the Instituto Mexicano del Seguro Social.

### Surgery

The rats were divided in two groups, one control group (CTR, *n* = 7), and one experimental group (EXP, *n* = 10). Both groups of rats were anesthetized under ketamine/pentobarbital anesthesia (60 mg/kg im, 20 mg/kg ip) and chronically implanted with bipolar, concentric electrodes in the MS (coordinates: 0.6 mm anterior to the bregma, 1.5 mm lateral to the midline, 15° from the vertical, and 6.8 mm ventral to the cranial surface), DG (coordinates 3.5 mm posterior to the bregma, 1.5 mm lateral to the midline, and 3.4 mm ventral to the cranial surface), CA1 (coordinates: 4.5 mm posterior to the bregma, 2.4 mm lateral to the midline, and 2.7 mm ventral to the cranial surface), and SUM (coordinates: 4.7 mm posterior to bregma, 0.2 mm lateral to the midline, and 8.7 mm ventral to the cranial surface); all coordinates were taken from the Atlas of Paxinos and Watson (1998). The electrodes were made of nichrome wire with a diameter of 60 μm fastened inside a stainless steel # 27 caliber cannula isolated with epoxy resin, with a small surface exposed at the tip. The electrodes were fixed to the skull with dental acrylic. Two screws were used, one placed in the frontal bone served as ground and the other placed in the posterior skull served to fix the implant. In the same surgery, rats in the EXP group received an intra-SUM infusion of 5 μg of 5,7-DHT (2 μg dissolved in 0.1 μl of 0.1% ascorbic acid in saline solution) at an infusion rate of 0.1 μl/min for 4 min. One injection was placed into the SUMn (4.7 mm posterior to the bregma, 0.2 mm lateral to the midline, and 8.24 mm ventral from the cranial surface) using a Hamilton syringe and an infusion pump. Thirty minutes before the 5-HT lesion, the rats received desipramine (30 mg/kg, ip) to protect the noradrenergic terminals. The rats of the CTR group only received an infusion of vehicle solution, similar in volume and rate to the EXP group.

### Behavioral test

Two weeks after the surgery, the rats were trained in a place-learning test using the Morris water maze. This maze consisted of a circular pool (1.5 m of diameter and 45 cm of height wall) filled with water made blue by adding gentian violet, which contained a submerged circular platform (9 cm of diameter) placed in a fixed position in one of the four virtual quadrants of the maze.

The rats were submitted to four daily trials during six consecutive days; each trial was initiated by placing the rat into the pool facing the wall in one of the quadrants (the starting quadrants were randomly chosen each day but were similar for all rats in one day). The trial continued until either the rat located the platform or 60 s elapsed. If the rat failed to locate the platform in this time, it was guided to the platform by the experimenter and left there for 15 s. After this time, the rat was retired and placed in a home cage during 2 min (inter-trial period) before beginning the next trial. On the seventh day, all rats received one 30 s probe trial that consisted of searching the maze after the escape platform had been removed. The behavioral tests were video recorded and stored on a computer for later analysis, when the escape latencies, distances traveled and swimming velocity achieved by the rats and also the distance swam for each quadrant in the probe trial were obtained. Recordings and analysis were performed using the Data-Wave Inc. software (VideoBench 5.1). The mean swim distances from the four daily trials as well as the mean daily latencies were compared. In the probe trial, the distance swam by the rats in each quadrant was obtained and compared.

### EEG records

Each training day the rats were connected to a commutator (Neuro-Tek, CA. IT,) using a cable with a male connector. The commutator was connected to one amplifier (Neurodata acquisition system, GRASS Mod 15, Astro Med Inc. 600 E. Greenwich Ave., W. Warwick, RI 02893, USA) and the EEG was digitalized to 1024 Hz with a DataWave Technologies data acquisition system, and the EEG was stored in a PC to be analyzed of line. A bipolar recording was taken using the nichrome wire as G1 and the cannula as G2 (A bipolar derivation was made using the G1–G2), the filters were set to 1–100 Hz, the EEG recording were synchronized to the VideoBench software, which tracked a small light-emitting diode attached to animal implant. A baseline recording was taken from the awake-immobile rat in the cage (60 s), and then, all time that the rat searched for the platform was recorded, including the final 15 s that the rats remained onto the escape platform. The data were imported into MATLAB (Mathworks, Inc.) (Delorme and Makeig, 2004) and the software EEGLAB was used to eliminate artifact by visual inspection.

The EEG from basal and searching conditions was submitted to the Fast Fourier Transform (FFT) and absolute power was obtained as the mean spectrum of 2-s samples, to ensure a resolution of 0.5 Hz, from 4 to 12 Hz. The relative power (RP) was obtained for each behavioral condition and 0.5 Hz of frequency as the percent of the total 4–12 Hz absolute power band. Comparisons were made of the RP in the range of 5–0 Hz, in each brain region, between days and frequency for each group (intra-group comparisons) and between day, group and frequency (inter-group comparison); using an ANOVA for repeated measures and paired *t*-test with a Bonferroni correction. Additionally, coherence values were computed for pairs of recording sites and compared in manner similar to the RP values. The analyses of both EEG power and coherence were conducted using custom programs adapted from Ken's MATLAB library written by Ken Harris and available at http://osiris.rutgers.edu/Buzsaki/software.

### HPLC

The serotonin content was determined using HPLC as follows, after the euthanasia of the animals, samples including SUMn were dissected from a slice containing the region of interest and a sample of the tissue was punched using a 25 G cannula. The tissue samples were homogenized in 1N HCl and centrifuged. The content of serotonin and 5HIAA (pg/mg of fresh tissue) of the supernatant was determined using a LiChroCart purospher star column (150 – 4.6, RP – 18 end caped, 5 mm, MERK KGa A, Darmstadt; Germany) with a mobile phase (pH 3.1) composed of citric acid (50 mM), H_3_PO_4_ (50 mM), EDTA (20 mg), octanesulfonic acid (120 mg/L), and methanol (8 %). The flow rate was 1.3 mL/min. An electrochemical detector (AtecLydenVT-03) with a work potential of 0.800 mV adjusted to the pH of the mobile phase was used. The data were compared using the Student *t*-test.

**Figure 1:**
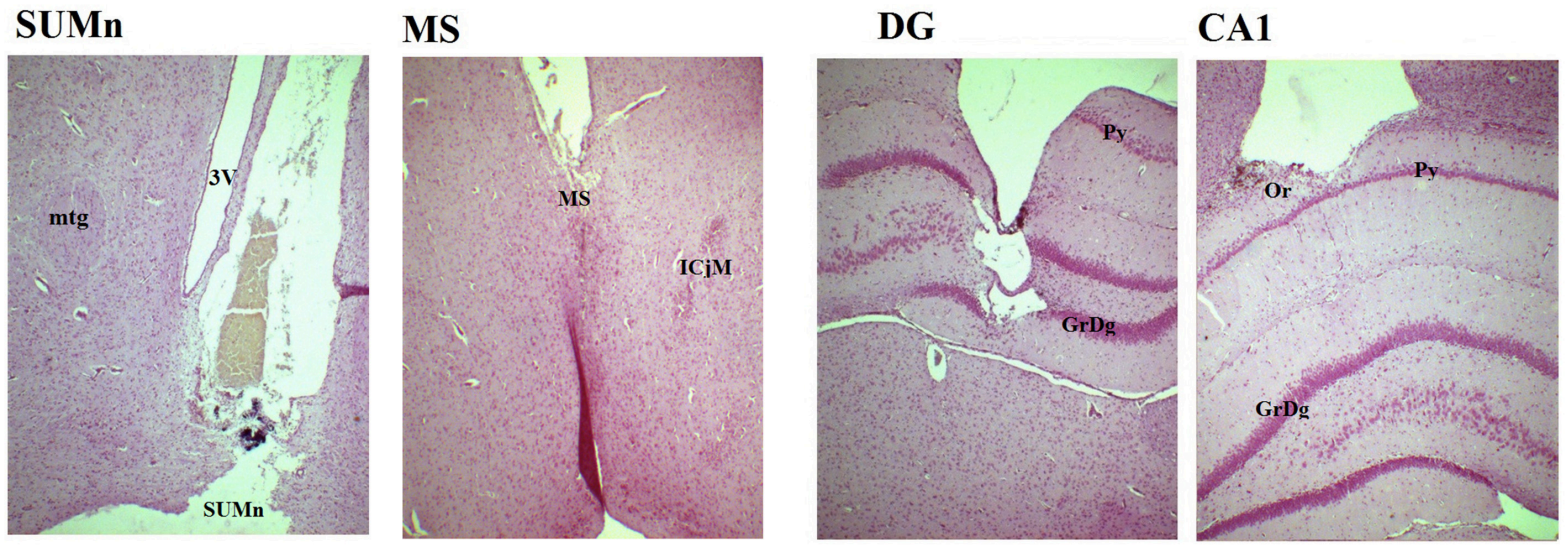
**Photomicrography of representative slices showing the position of the electrodes in the four regions**. In the SUMn note that tract of the electrode reaches the zone in which the tissue was punched out for HPLC measures. SUMn, supramammillary nucleus; mtg, mamillotegmental tract; 3V, 3rd ventricle; MS, medial septum; ICjM, Major island of Calleja; DG, dentate gyrus; GrDg, granular layer of dentate gyrus; Py, pyramidal layer of CA1; CA1, field CA1 of hippocampus; Or oriens layer. Magnification 4X.

The SUMn was visually inspected to verify the electrode position, during the dissection of the tissue for HPLC. The tract of the electrode in the remaining tissue after the dissection of SUMn for HPLC and the position of the other electrodes was verified using a light microscope after the brain was sliced at 5 μm and the slices were stained with cressyl violet (Figure 1). After histological verification of the position of the electrodes in the MS, the DG and the CA1; the EXP group of rats included only those rats with reductions of serotonin greater than 50% from the CTR group mean content in the SUMn; thus, four rats were excluded because they showed a reduction of serotonin less than 50%, and the EXP group included 6 rats in the final analysis.

## Results

### Serotonin content

**Figure 2:**
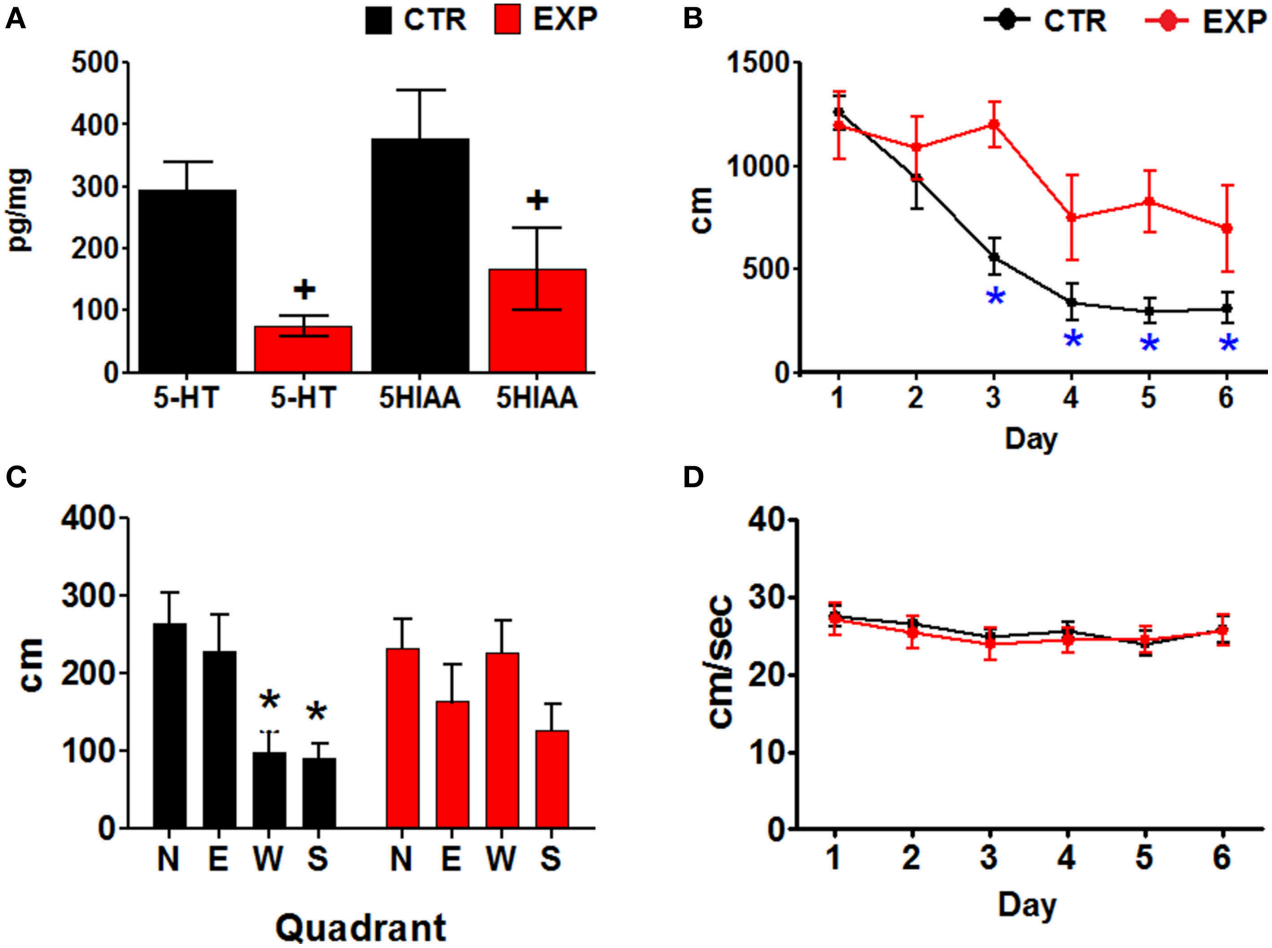
**(A)** Serotonin and 5-HIAA concentrations. Mean ± SEM, *p* < 0.05. **(B)** Distances traveled by the two groups of animals, through the training days. Mean ± SEM. ^*^Day 1 vs. subsequent days, *p* < 0.001. **(C)** Distance traveled in each quadrant during the probe test of the two groups of rats. The escape platform was placed in the north quadrant during the training. North (N), South (S), East (E), and West (W) quadrants. Mean ± SEM. ^*^N quadrant vs. all other quadrants; +, group CTR vs. EXP, *p* < 0.05. **(D)** Swimming velocities displayed by the two groups of animals through the training days. Mean ± SEM.

The EXP group had significantly lower serotonin (5-HT) and 5-hydroxyindoleacetic acid (5-HIAA) concentrations that the CTR group (Paired one tailed *t* = 4.274, *df* = 5, *p* = 0.004 and *t* = 7.293, *p* = 0.0004, *df* = 5; for 5-HT and 5HIAA, respectively) (Figure 2A).

### Behavior

Escape latencies were compared between training days within the two groups of animals using a Friedman ANOVA, and a *post-hoc* Wilcoxon test. The CTR group significantly reduced their escape latencies (X${}_{\text{r}}^{2}$= 23.245, *P* > 0.001), by day 3–6 (*p* = 0.018); whereas the EXP group only significantly reduced their escape latencies (X${}_{\text{r}}^{2}$ = 11.429, *P* = 0.044), on day 6 (*p* = 0.028). Intergroup comparisons (Mann Whitney U test) showed both a main effect (∑R_x_ = 391, *P* < 0.001) and differences in the escape latencies on days 3 (*p* = 0.004), and 5 (*p* = 0.003) with a bias toward day 4 (*p* = 0.063); the escape latencies were longer for the EXP than for the CTR group (Data not showed).

Intra-group comparisons of the distances traveled by the rats were made using an ANOVA for blocks and Tukey *post-hoc*; the CTR group significantly reduced their distances traveled [*F*_(5, 30)_ 24.112, *p* < 0.001], on days three to six of training (*p* < 0.001). The EXP group did not show significant reduction of distance traveled over the training days [*F*_(5, 25)_ = 2.018, *p* = 0.111]. Inter-group comparisons using two factors, group and day of training, were made using an ANOVA for repeated measures. The distances traveled by the EXP group were higher than the distances traveled by the CTR group [*F*_(1, 11)_ = 11.232, *p* = 0.006, main effect], however, there was no significant interaction of day and group [*F*_(5, 55)_ = 2.242, *p* = 0.062] (Figure 2B). The swimming velocities were compared similarly to the distances, but no changes over the training days were observed for the CTR [*F*_(5, 30)_ = 1.472, *P* = 0.228] or the EXP [*F*_(5, 25)_ = 1.981, *P* = 0.116] groups (Figure 2D).

Finally, the distance traveled in each quadrant during the probe trial (day seven) was compared between quadrants and groups, using a Two-Way ANOVA (group and quadrant). No significant differences between groups were observed [*F*_(3, 48)_ = 2.452, *P* = 0.074]. However, the CTR group swam significantly different distances between quadrants [*F*_(3, 24)_ = 6.285, *p* = 0.002]; the distance on the quadrant that had contained the platform in the training (N) was higher than in the S and W quadrants. The EXP group of animals swam similar distances in all quadrants (Figure 2C).

### Theta activity

**Figure 3:**
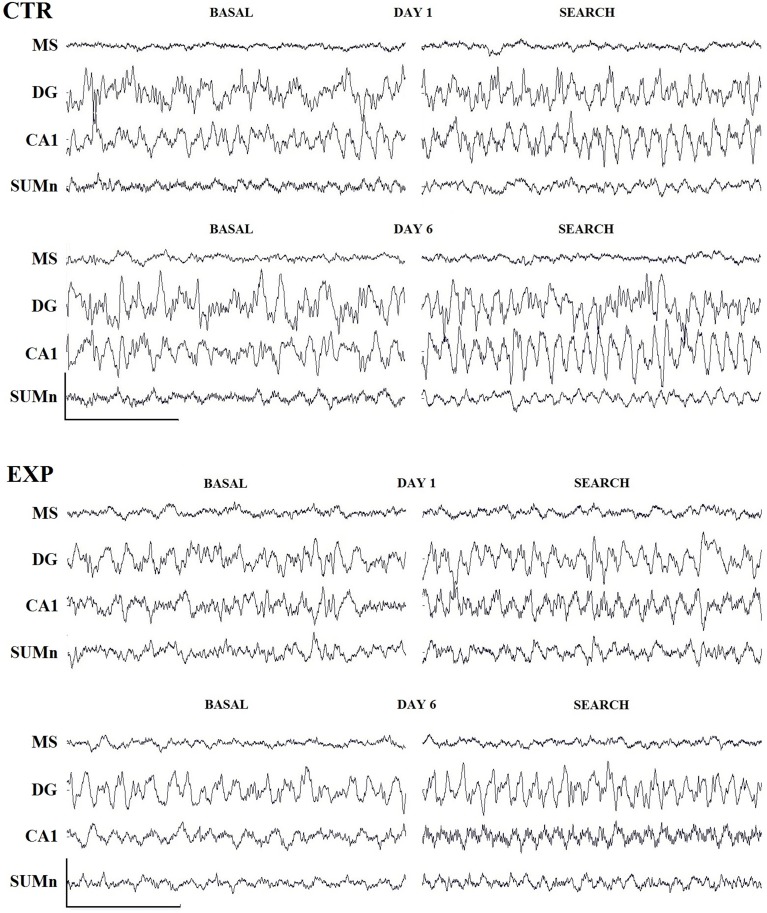
**Row traces of one representative rat from each group**. Three second samples recorded during basal conditions (awake, wet rat in a holding cage) and during the search for the platform on days 1 and 6 of training. Cal. 500 mV/1 s. Abbreviations are as in the text.

The row EEG from the four regions recorded, under basal conditions (awake, immobile, wet rat) in the cage and during the searching for the platform on days one and six of representative rats, is shown in Figure 3. The natural logarithm (nl) of the absolute power of the theta band from each cerebral region and group was compared by day and frequency using Two-Way ANOVA. No significant differences were observed in any group regarding this comparison (data not shown). Intergroup comparisons of the absolute power of the nl recorded from each cerebral region were performed using ANOVA for repeated measures of two factors (group and frequency), with days as a repeated measure; no significant differences were observed for any of the regions studied.

Relative power, expressed as a percentage of the contribution of each specific frequency to the total power of the theta band, had a beneficial effect of reducing the inter-subject variance. In addition, it is possible that the changes associated with learning on EEG could be sufficiently subtle to reflect absolute power changes; moreover, the consequences of a reduction of one neurotransmitter in one discrete nucleus from the SAS could be quite subtle and could induce changes in the expression of absolute power in the theta band. Thus, more subtle changes were expected than those observed in studies in which the cerebral reduction of serotonin or RM lesions was induced. Using this rationale, in previous studies, learning-related changes were observed in the relative power of the theta activity recorded in CA1 during the training of rats in the Morris water maze (8–10).

**Figure 4:**
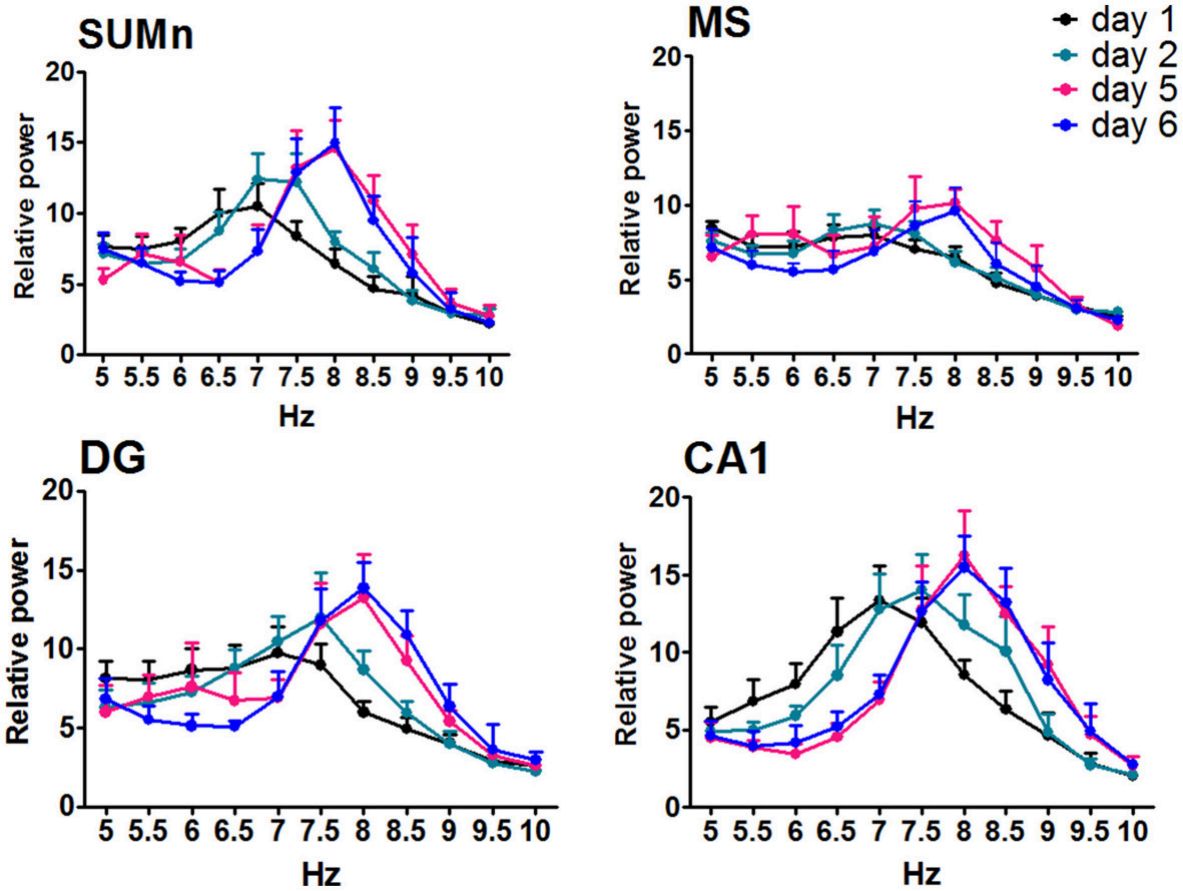
**Relative power in the theta band (5–10 Hz) recorded in the four regions of the CTR group during the searching of the platform across days of training**. Only days 1, 2, and 5, 6 are showed. Mean ± SEM. Significant differences are listed in Table 1. *p* < 0.05.

The relative power (RP) of each cerebral region of the CTR group was compared by day and frequency using a Two-Way ANOVA for repeated measures. The RP recorded in the SUMn for the CTR group changed across the training days [*F*_(50, 330)_ = 2.977, *p* < 0.0001]. The RP for high frequencies (7.5–8.5 Hz) RP increased with the training days whereas low frequencies (6.5 and 7 Hz) decreased when compared with the first and second days. RP from MS showed significant changes across the training days [*F*_(50, 330)_ = 1.477, *p* = 0.025]; particularly an increase for the 8 Hz frequency the lasts days of training. The RP from the DG showed increased theta activity over the course of the training days [*F*_(50, 330)_ = 2.689, *p* < 0.0001] for the 7–8.5 Hz frequencies. Finally, the RP of the theta activity recorded in the CA1 showed changes with regard to training days [*F*_(50, 30)_ = 2.729, *p* < 0.0001], and the RP for the 6.5 and 7 Hz frequencies was reduced, whereas the RP for 8.0 and 8.5 Hz increased over the training days. Figure 4 shows the RP only for days 1, 2, 5, and 6 when the differences between RP were maximal, and Table 1 shows the significant differences between all of the training days from the four regions. Thus, the RP in the higher frequencies (7–5–10 Hz) increased across the training days in the different regions, and some regions showed a concomitantly reduction in low frequencies RP (6.5–7 Hz).

**Table 1:** **Comparison between training days of the relative power recorded during the searching for the platform in the Morris water maze task in the CTR group**.

**Hz\Day**	**1**	**2**	**3**	**4**	**5**	**6**	**Region**
6.5	10.00 ± 1.71	8.74 ± 1.32	6.97 ± 0.92	6.78 ± 0.97	5.16 ± 0.85^A^	5.11 ± 0.85^A^	SUM
7	10.49 ± 1.62	12.40 ± 1.85	11.25 ± 1.98	10.17 ± 2.13	7.30 ± 1.84^B^	7.31 ± 1.53^B^	
7.5	8.40 ± 0.89	12.2 ± 2.02	14.23 ± 2.30^A^	13.98 ± 3.21^A^	13.23 ± 2.59^A^	12.87 ± 2.40^A^	
8	6.45 ± 1.00	7.98 ± 0.73	10.19 ± 0.96	11.73 ± 1.67^A^	14.57 ± 1.97^ABC^	14.96 ± 2.47^ABC^	
8.5	4.70 ± 0.84	6.07 ± 1.15	7.02 ± 1.70	8.55 ± 2.30	10.86 ± 1.80^AB^	9.48 ± 1.74^A^	
8	6.50 ± 0.72	6.13 ± 0.73	6.23 ± 0.86	7.39 ± 0.60	10.13 ± 0.91^A^	9.62 ± 1.54^A^	MS
7.5	11.90 ± 1.59	14.04 ± 2.29	16.47 ± 3.02^A^	13.08 ± 3.09	12.73 ± 2.84	12.60 ± 1.91	DG
8	8.51 ± 0.97	11.76 ± 1.95	12.64 ± 2.73^A^	10.62 ± 1.57	16.27 ± 2.87^A^	15.48 ± 2.01^ABCD^	
8.5	6.32 ± 1.20	10.05 ± 2.23	9.01 ± 2.26	8.43 ± 2.15	12.51 ± 1.74^A^	13.20 ± 2.20^ABD^	
6.5	11.32 ± 2.17	8.49 ± 1.96	6.43 ± 1.36	6.66 ± 1.28	4.55 ± 0.89	5.19 ± 0.96^A^	CA1
7	13.36 ± 2.25	12.75 ± 2.33	12.15 ± 2.40	9.74 ± 1.68	6.93 ± 1.13^BC^	7.29 ± 1.24^AB^	
8	8.51 ± 0.97	11.76 ± 1.95	12.64 ± 2.73	10.62 ± 1.57	16.27 ± 2.87^AD^	15.48 ± 2.012^A^	
8.5	6.32 ± 1.20	10.05 ± 2.23	9.01 ± 2.26	8.43 ± 2.15	12.51 ± 1.74^A^	13.20 ± 2.20^A^	

ANOVA including the 6 days of training was significant. Values are the mean ± SEM. A, B, C, and D show significant differences compared with days 1, 2, 3 and 4, respectively. P < 0.05.

**Figure 5:**
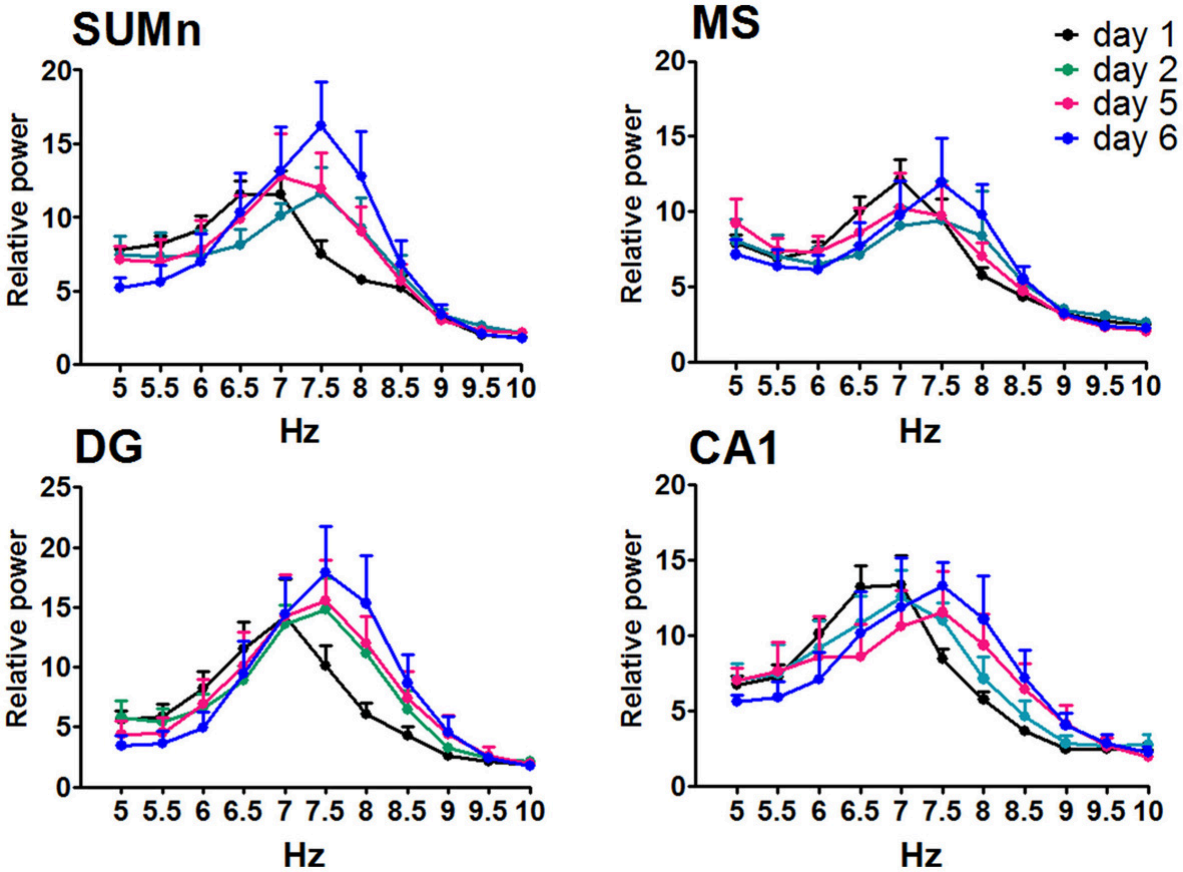
**Relative power in the theta band (5–10 Hz) recorded in the four cerebral regions of the EXP group during the searching of the platform across training days**. The ANOVA was significant only when compared the days 1, 2, 5, and 6. Mean ± SEM. Significant differences are listed in Table 2. *p* < 0.05.

The RP recorded in the four cerebral regions from the EXP group did not show significant effects of training across training days [*F*_(50, 275)_ = 1.272, *p* = 0.1181 for MS; *F*_(50, 275)_ = 1.010, *p* = 0.4622 for DG; *F*_(50, 275)_ = 1.272, *p* = 0.1178; and *F*_(50, 275)_ = 1.368, *P* = 0.0616 for SUMn]. However, there were days in which the information processing was putatively different, that is, acquisition of information is prominent on days 1 and 2, whereas the consolidation and recovery of memory is prominent on days 5 and 6; therefore, an ANOVA including only days 1, 2, 5, and 6 for the EXP group was performed to determine whether the differences in processing would be expressed as differences in EEG in this group. The SUMn RP showed significant changes across training days when only the mentioned days were considered [*F*_(30, 165)_ = 2.194, *P* = 0.0009]; with increases in the RP for the 7.5 and 8 Hz frequencies. In addition, in the CA1 region, the RP showed significant changes across days [*F*_(30, 165)_ = 1.750, *p* = 0.0146] for the frequencies 6.5, 7.5, and 8 Hz (Figure 5), the Table 2 shows the significant differences in the two regions. In summary, the EXP group had minimal changes related to the process of leaning evident only when the comparisons included only the days 1, 2, 5, and 6. Moreover, the increased RP observed was limited to SUM and CA1 and occurred at 7.5 and 8 Hz, whereas no change was evident in this group at 8.5 Hz.

**Table 2:** **Comparison between training days of the relative power recorded during the searching for the platform in the Morris water maze task in the EXP group**.

**Hz\Day**	**1**	**2**	**3**	**4**	**5**	**6**	**Region**
7.5	7.49 ± 0.93	11.60 ± 1.81	12.96 ± 2.20	10.18 ± 1.75	11.92 ± 2.47	16.25 ± 2.96^AB^	SUM
8	6.72 ± 0.32	9.28 ± 2.07	9.35 ± 1.84	9.31 ± 2.80	9.01 ± 1.71	12.81 ± 3.01^A^	
6.5	13.22 ± 1.45	10.86 ± 1.76	10.88 ± 1.94	9.09 ± 2.50	8.60 ± 2.15^A^	10.18 ± 2.75	CA1
7.5	8.46 ± 0.66	10.99 ± 1.20	11.36 ± 2.13	11.13 ± 1.70	11.57 ± 2.68	13.30 ± 1.53^A^	
8	5.79 ± 0.47	7.16 ± 1.42	8.26 ± 1.94	8.13 ± 1.70	9.41 ± 2.68	11.14 ± 2.83^A^	

ANOVA including the days 1, 2, 5, and 6 of training was significant. Values are the mean ± SEM. A, B, C, and D, show significant differences compared with days 1, 2, 3, and 4; respectively. P < 0.05.

**Figure 6:**
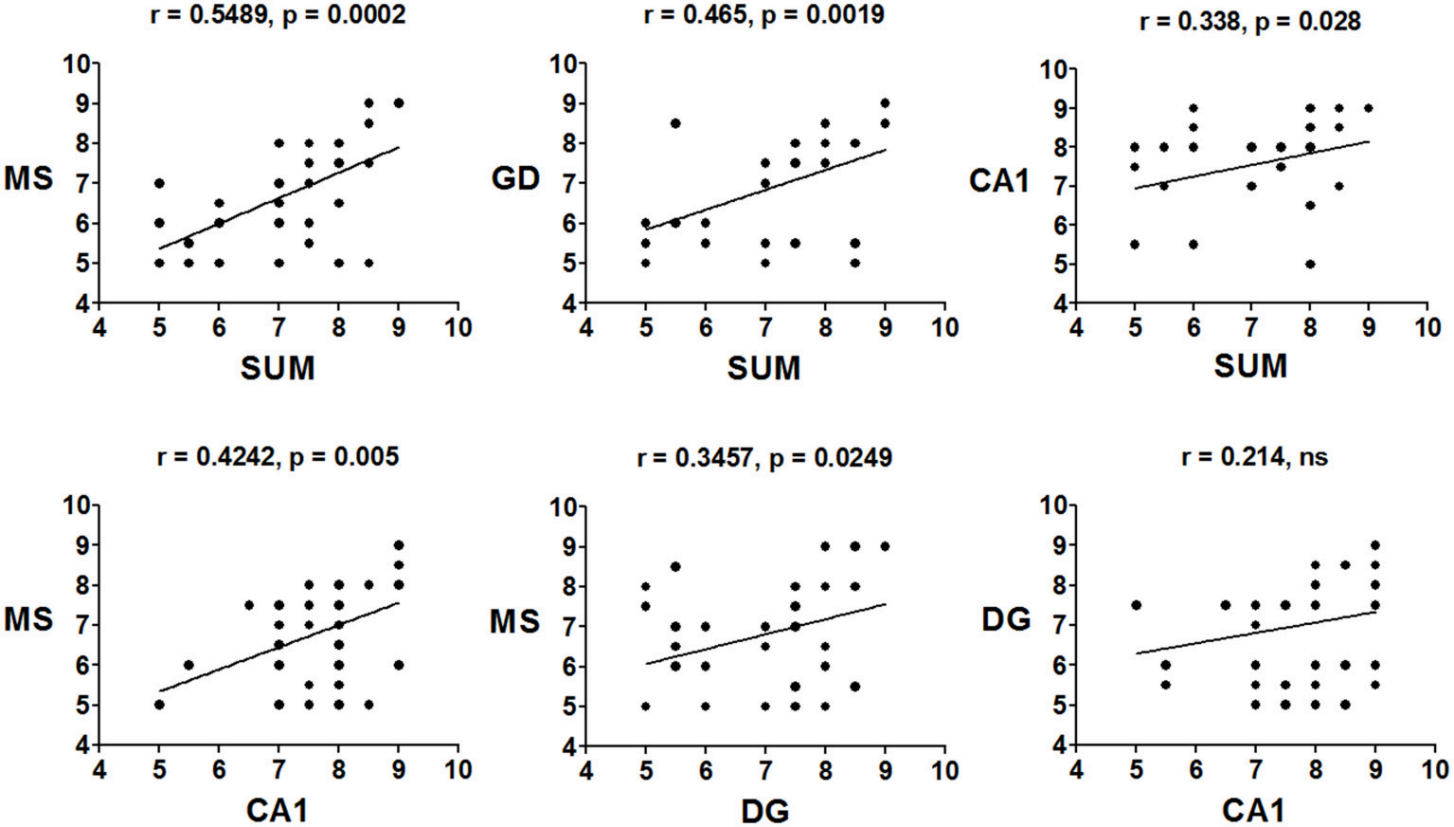
**Correlations of the mean frequency peak of the RP in the theta band (5–10 Hz) between cerebral regions, across training days, in the CTR group**. Significant positive correlations between the three regions (MS, SUMn and Hippocampus), but not within the hippocampus (DG and CA1) were observed (ns, no significant).

**Figure 7:**
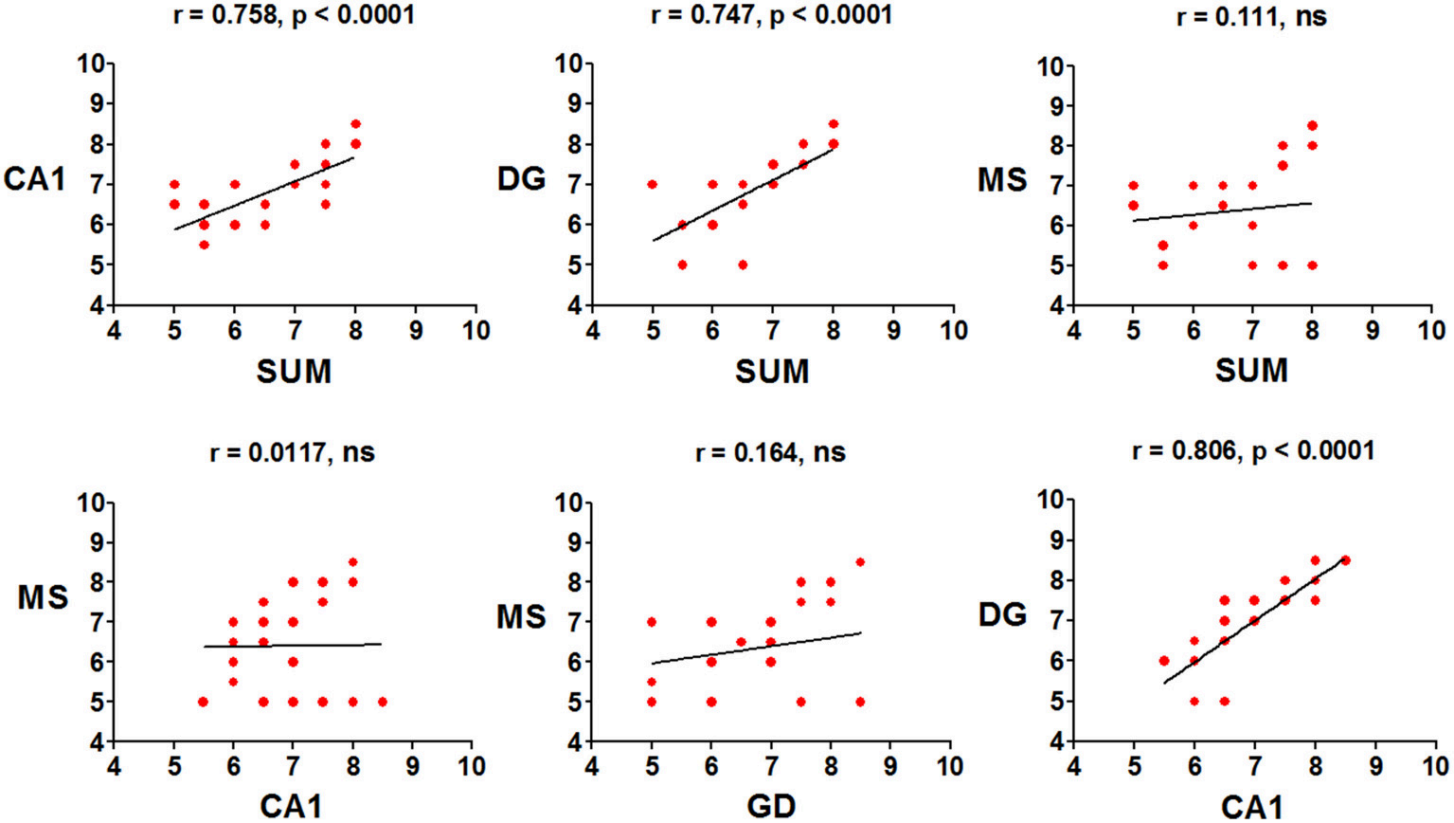
**Correlations of the mean frequency peak of the RP in the theta band (5–10 Hz) between recorded regions, across training days, in the EXP group**. Significant positive correlations between the SUMn and the hippocampus and within the hippocampus (DG and CA1), were observed. No correlation between the MS and the other two regions occurred in this group (ns, no significant).

The mean peak frequency of each day of training from the four daily trials was obtained, and intra-group comparisons were made using ANOVA for blocks. The CTR group significantly increased the peak frequency in the SUMn [*F*_(5, 30)_ = 60.061, *p* < 0.001]; however, paired comparisons (Tukey's test) did not show significant differences compared with day 1. Additionally, the peak frequency in the DG increased with the day of training [*F*_(5, 30)_ = 4.611, *p* = 0.003]; the peak frequency increased on days 5 (*p* = 0.004) and 6 (*p* = 0.034) compared with the first day of training. The EXP group did not show increase in the peak frequency across training days in any region. Finally, the Pearson correlation of the peak frequency between pairs of regions across all training days was calculated, to establish whether the changes in peak frequency were similar between them, both in control conditions and after serotonin depletion in the SUMn. In the CTR group, the peak frequencies were positively and significantly correlated between the SUMn and hippocampus (both the CA1 and the DG), between the MS and the hippocampus (both the CA1 and the DG), and between the SUMn and the MS; although no significant correlation in peak frequency was observed between the CA1 and the DG (Figure 6). The EXP group, however, showed high positive correlations between peak frequencies of the SUMn and the hippocampus (both the CA1 and the DG), but no significant correlations were observed between the MS and the hippocampus nor between the SUMn and the MS; moreover, this group showed significant correlation in the peak frequency within the hippocampus (the DG and the CA1) (Figure 7). These results imply that the peak frequency of the EEG in the CTR group is related in the three structures (SUMn, MS, and hippocampus), but no relation exists within the hippocampus; in contrast, in the EXP group a closer relation occurs between the SUMn and the hippocampus with a disengagement of MS.

**Figure 8:**
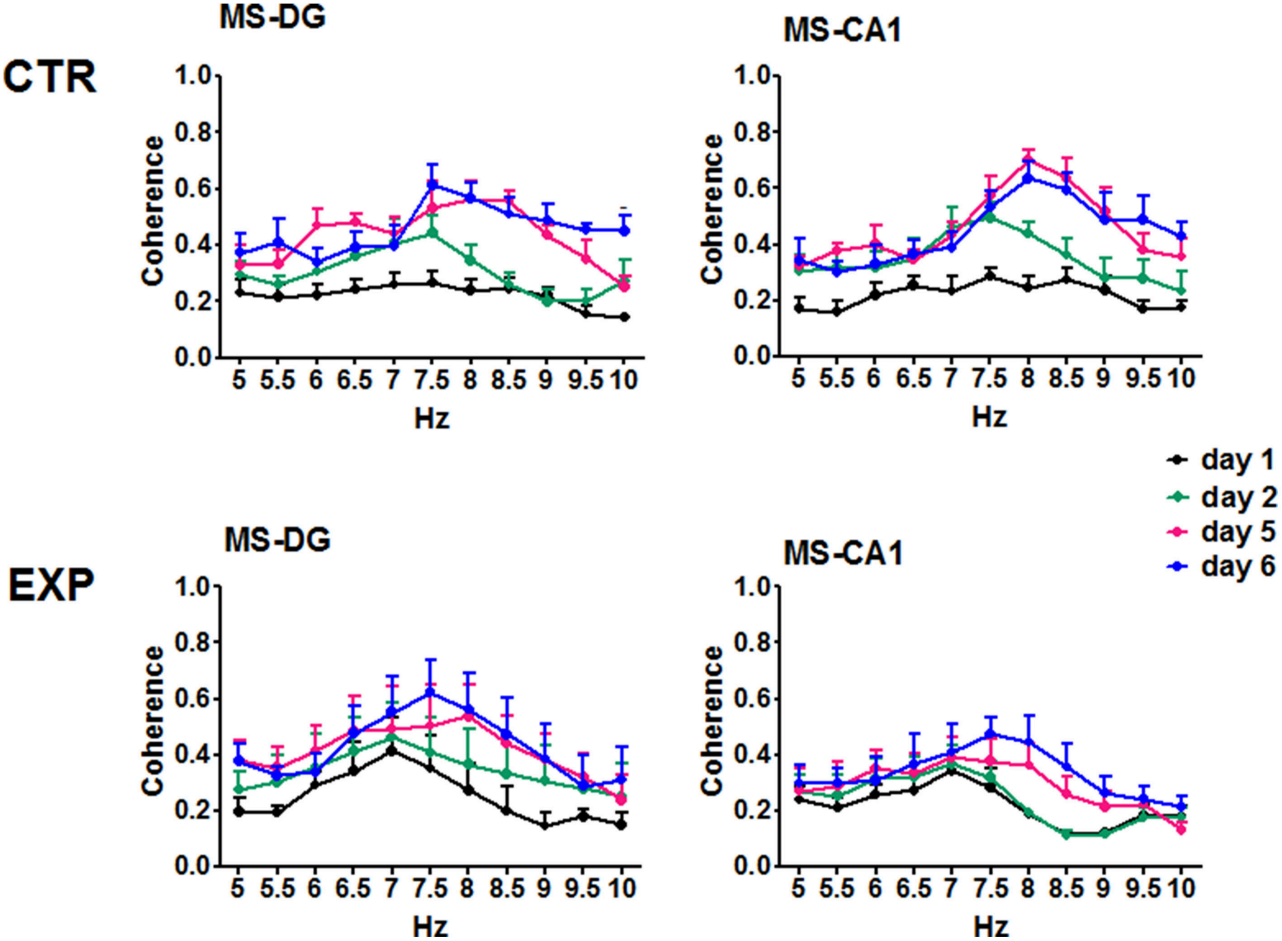
**Coherences of the EEG recorded in the MS, DG and CA1 in the two groups across training days**. For the CTR group the ANOVA was significant only considering the days 1, 2, 5, and 6. Only CTR group showed increased coherence between MS-DG and MS-CA1. Mean ± SEM. Significant differences are listed in Table 3. *p* < 0.05.

Coherence was compared between training days and frequency using ANOVA for repeated measures, for each group. In the CTR group no significant change was observed in the coherence between regions regarding the training days when all 6 days of training were included [*F*_(50, 330)_ = 1.052, *p* = 0.3852 for MS-DG; *F*_(50, 330)_ = 1.335, *p* = 0.741 for MS-CA1; *F*_(50, 330)_ = 0.8018, *p* = 0.8281 for MS-SUMn; *F*_(50, 330)_ = 1.036, *p* = 0.4131 for DG-CA1; *F*_(50, 330)_ = 0.8514, *p* = 0.7519 for DG-SUMn; and *F*_(50, 330)_ = 1.030, *p* = 0.4236 for CA1-SUMn]. Using the same rationale used in the RP comparisons, ANOVA tests were applied for the days 1, 2, 5, and 6 of training, and significant effects of the training days over time were thus observed for the coherence between MS-DG [*F*_(30, 198)_ = 1.788, *p* = 0.0104] and MS-CA1 [*F*_(30, 198)_ = 1.609, *p* = 0.0300]. Paired comparisons (t-test with Bonferroni correction) showed significant increased coherence for both MS-DG MS-CA1 coherence on days 5 and 6 principally in the higher frequencies of the theta band, the coherences of MS-CA1 and MS-DG, for the CTR group are presented in the Figure 8, means and significant differences are presented in the Table 3.

**Table 3:** **Comparison between training days of the coherence between the EEG recorded during the searching for the platform in the Morris water maze task in the CTR group**.

**Hz\Day**	**1**	**2**	**3**	**4**	**5**	**6**	**Regions**
5.5	0.214 ± 0.043	0.260 ± 0.027	0.311 ± 0.056	0.376 ± 0.070	0.331 ± 0.050^A^	0.409 ± 0.084	MS-DG
6	0.222 ± 0.037	0.306 ± 0.029	0.295 ± 0.053	0.352 ± 0.040	0.469 ± 0.059^A^	0.338 ± 0.048	
6.5	0.241 ± 0.036	0.360 ± 0.085	0.336 ± 0.055	0.290 ± 0.035	0.480 ± 0.029^A^	0.391 ± 0.053	
7.5	0.266 ± 0.038	0.442 ± 0.062	0.405 ± 0.074	0.438 ± 0.100	0.530 ± 0.094^A^	0.613 ± 0.068^A^	
8	0.238 ± 0.039	0.344 ± 0.056	0.374 ± 0.089	0.465 ± 0.082	0.561 ± 0.062^AB^	0.565 ± 0.056^AB^	
8.5	0.246 ± 0.034	0.259 ± 0.043	0.368 ± 0.068	0.429 ± 0.063	0.558 ± 0.029^AB^	0.510 ± 0.056^AB^	
9	0.221 ± 0.027	0.198 ± 0.043	0.317 ± 0.072	0.332 ± 0.060	0.433 ± 0.035^AB^	0.486 ± 0.060^AB^	
9.5	0.156 ± 0.028	0.200 ± 0.043	0.228 ± 0.050	0.351 ± 0.067	0.349 ± 0.067^A^	0.454 ± 0.020^AB^	
10	0.144 ± 0.017	0.271 ± 0.073	0.195 ± 0.044	0.371 ± 0.058	0.251 ± 0.038	0.448 ± 0.056^A^	
5.5	0.159 ± 0.038	0.319 ± 0.062	0.295 ± 0.084	0.349 ± 0.067	0.377 ± 0.026^A^	0.301 ± 0.035	MS-CA1
7	0.235 ± 0.049	0.461 ± 0.071^A^	0.533 ± 0.050	0.411 ± 0.084	0.432 ± 0.045	0.389 ± 0.055	
7.5	0.288 ± 0.028	0.494 ± 0.055	0.611 ± 0.045	0.529 ± 0.088	0.572 ± 0.070^A^	0.530 ± 0.060^A^	
8	0.246 ± 0.040	0.437 ± 0.039	0.563 ± 0.048	0.532 ± 0.075	0.700 ± 0.037^AB^	0.635 ± 0.059^A^	
8.5	0.275 ± 0.043	0.363 ± 0.057	0.458 ± 0.058	0.494 ± 0.076	0.634 ± 0.076^AB^	0.594 ± 0.062^AB^	
9	0.238 ± 0.045	0.280 ± 0.069	0.451 ± 0.092	0.455 ± 0.080	0.517 ± 0.085^AB^	0.486 ± 0.096^AB^	
9.5	0.168 ± 0.028	0.279 ± 0.065	0.328 ± 0.088	0.385 ± 0.069	0.379 ± 0.059^A^	0.489 ± 0.083^A^	
10	0.175 ± 0.023	0.235 ± 0.066	0.440 ± 0.071	0.375 ± 0.060	0.357 ± 0.061	0.429 ± 0.051^A^	

ANOVA including the days 1, 2, 5 and 6 of training was significant. Values are the mean ± SEM. A and B, show significant differences compared with days 1, and 2; respectively. P < 0.05.

The EXP group coherences were also compared considering day and frequency using ANOVA for repeated measures. No significant effects of the training in the inter-region coherences were observed for the EXP group when all six training days were considered [*F*_(50, 275)_ = 0.5824, *p* = 0.9887 for MS-DG; *F*_(50, 275)_ = 0.6685, *p* = 0.9567 for MS CA1; *F*_(50, 275)_ = 0.4951, *p* = 0.9983 for MS-SUM; *F*_(50, 275)_ = 0.8108, *p* = 0.8130 for DG-CA1; *F*_(50, 275)_ = 0.5119, *p* = 0.9974 for DG-SUM; *F*_(50, 275)_ = 0.4132, *p* = 0.9998 for CA1-SUM], nor when only days 1, 2, 5, and 6 were considered. Coherences of MS-DG and MS-CA1 EEG, from the EXP group are shown in Figure 8.

In order to know if a shift occurred through the training days in the frequency in which the peak of coherence occurred (frequency of the coherence peak, FCP), the FCP and the magnitude of the peak of coherence were compared between days of training in both groups of animals. The CTR group MS-DG, MS-CA1, and DG-SUMn FPCs, showed increases, whereas the EXP group did not show changes. In the magnitude of the peak of coherence all pairs of regions showed increases in the CTR group, whereas in the EXP group only MS-CA1, MS-SUMn, and DG-SUMn showed increase with the training. Intergroup comparison showed higher FCP in CA1-SUM and MS-CA1, and higher magnitude of the peak for MS-CA1 for the CTR group (Figure S1). Thus, the EEG of the MS and hippocampus increased in coherence with the establishment of the memory in the CTR group but not in the EXP group.

**Figure 9:**
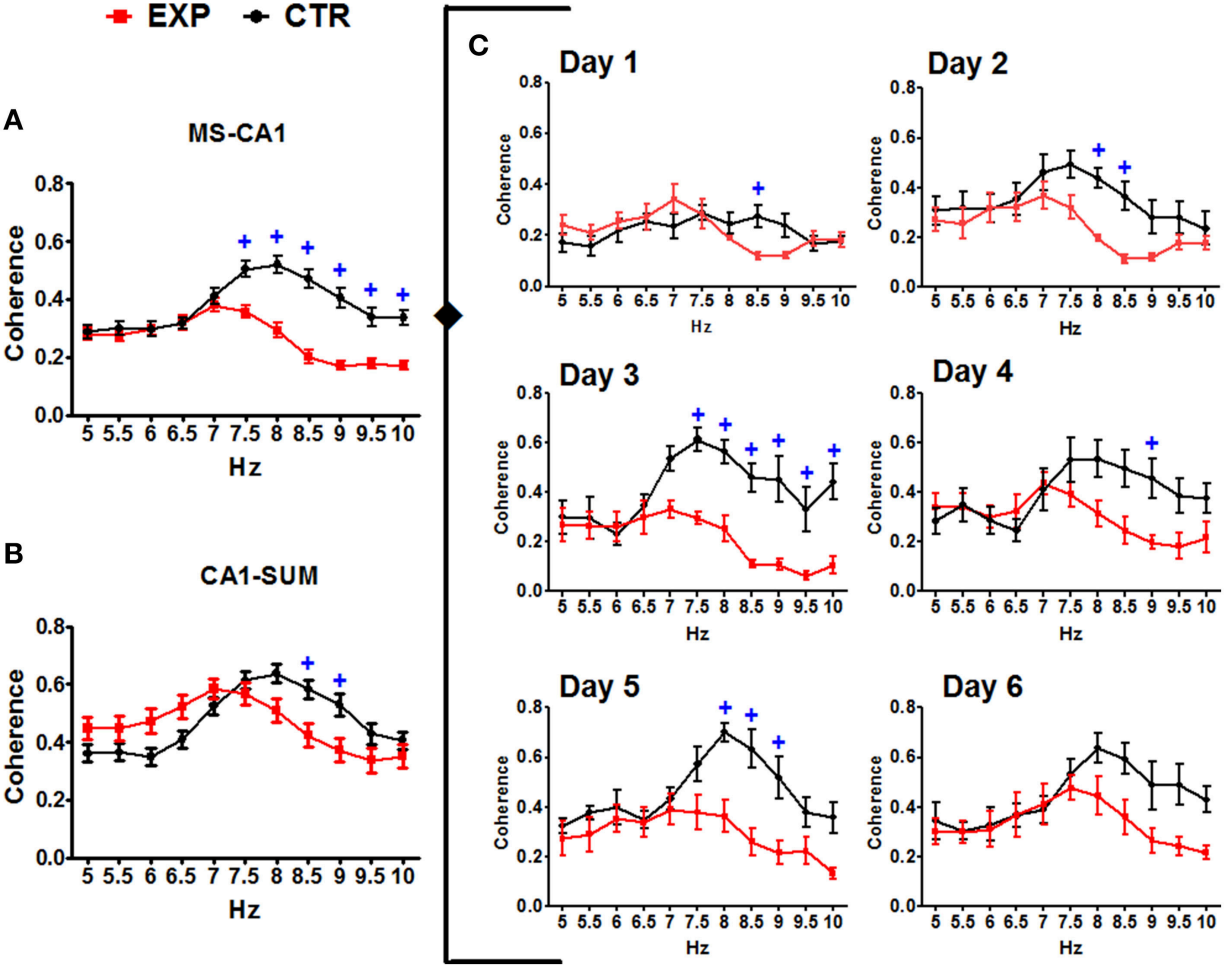
**Coherences between the EEG recorded in the MS-CA1 and CA1-SUMn, in the two groups. Inter-group comparisons in the MS-CA1 coherence (A) and in CA1-SUMn coherence (B), were observed (main effect)**. MS-CA1 coherence was also different through the days of training (C). Mean ± SEM. +, Group CTR vs. group EXP. *p* < 0.05.

Inter group comparisons of the coherence between pairs of regions were made using an ANOVA for repeated measures considering the factors group and frequency as independent and the training days (1–6) as repeating. MS-CA1 coherences showed significant effects both for the interaction of frequency and group [*F*_(1, 138)_ = 17.726, *p* < 0.0001] and for the interaction of frequency, group and day [*F*_(5, 690)_ = 2.478, *p* = 0.031]. Paired comparisons between frequency and group showed higher coherence between MS-CA1 regions for the CTR group from 7.5 to 10 Hz compared with the EXP group. When paired comparisons considering the training day were made, the EXP group showed lower coherences across days one to five, on day 1 in the 8.5 Hz frequency, on day 2 in the 8 and 8.5 Hz frequencies, on day 3 in the 7.5–10 Hz frequencies, on day 4 in the 9 Hz frequency and on day 5 in the 8–9 Hz frequencies. Moreover, CA1-SUMn coherences showed a significant effect of the interaction between frequency and group [*F*_(1, 138)_ = 7.182, *p* = 0.008], paired comparisons showed lower coherences for the EXP group in the 8.5 and 9 Hz frequencies than for the CTR group (Figure 9). The EXP group differed from the CTR group in both the pattern and degree of coherence.

## Discussion

The participation of the SUMn in place learning and memory has been controversial, with some studies reporting no or minimal effect on spatial learning and memory, after inactivation or inhibition of SUMn, and other studies implying the participation of the SUMn in retention and consolidation of spatial reference memory (Pan and McNaughton, 1997; Santin et al., 2003; Shahidi et al., 2004a; Aranda et al., 2008; Gutiérrez-Guzman et al., 2012). It was reported that lidocaine infusion into the SUMn did not affect the acquisition of an avoidance task although retention was impaired when lidocaine was infused before training. Additionally, post-training infusion caused impairments in consolidation of memory in this task (Shahidi et al., 2004b). Shahidi et al. (2004b) evaluated the effects of SUMn inactivation on spatial reference memory and spatial working memory using a Morris maze with a training schedule of 8 daily trials for 3 days. The authors did not observe alterations in reference memory when inactivation was performed before training; this implies that participation of SUMn is not crucial in the acquisition of spatial reference information. However, the author observed deficiencies when the SUMn was inactivated after the training buy prior to the probe trial. The present results showed severe impairment in spatial reference memory after SUMn serotonin depletion such that no significant reduction in the distances traveled was achieved by this group, and although the animals eventually attained a significant reduction in their escape pathways, this group searched similarly throughout the four quadrants in the probe trial. It could be interpreted that no learning was achieved by these animals based on the absence of reductions in the path lengths over the six training days; however, it was evident from the latencies in escape and the lengths of the pathways that these animals performed intermittently, presenting good performance on one trial or day and on the next trial or day and performing as badly as on the first day of training (see Figure 2). Thus, in spite of the severe deficiencies, the serotonin depletion did not appear to completely impair the acquisition of the spatial reference information. The deficiencies in spatial learning in the present work could be related to the impaired consolidation across days of training, according to the Shahidi results; however, some information could be acquired, although it is uncertain if the SUMn serotonin-depleted rats would have reached the control performance level with more training days. Together with the previous report in which serotonin depletion of both the SUMn and the PHn induced deficient but not absent place learning, these results support the participation of SUMn in spatial memory consolidation. Although we cannot exclude the participation of the PH, the present results show that only SUMn serotonin depletion produced deficiencies in place learning, similar to the results observed after the simultaneous serotonin depletion of the two nuclei, supporting a principal role for the SUMn in place learning.

In the present work, changes in RP were observed in CA1 theta activity, consistent with the previous studies. In addition, in the present work, evidence showed similar changes in the SUMn, that is, the decrease in RP at low frequencies (6–7 Hz) and the increase in RP at high frequencies (7.5–8.5 Hz) across training days; these changes were possibly related to the consolidation of spatial information. Furthermore, extending the brain regions previously recorded, the RP also increased in the DG (8 and 8.5 Hz), although no reduction of low frequencies was observed in the RP on this region comparing all days of training. In order to explore a possible difference in the last day compared with the first, in low frequencies, these 2 days were compared, and significant reduction was observed at 6 and 6.5 Hz frequencies the day six with respect to the first day of training. Finally, MS RP increased only in the 8 Hz frequency, this last could be an effect of broadening of the spectrum. Minor changes were evident in the EXP group only when days 1, 2 and 5, 6 were compared.

Recently a reduction in CA1 theta activity was reported in rats exposed to unexpected environmental changes, whereas they developed foraging activity (Jeewajee et al., 2008). As mentioned previously, in earlier studies assessing the relationship of theta activity with place learning ability, it was reported that the RP of the high frequency theta band (6.5–9.5 Hz) recorded in the CA1 region increased over training days in intact rats trained in the Morris spatial test, and these increases were absent in rats trained in egocentric and cue versions of the task and in aged inefficient rats (8–10). If the increased RP at high frequencies observed in the present work were due to the novelty effect for exposure of the rats to the new environment, the three groups of animals trained in the aforementioned study would have shown similar changes in their theta RP throughout the training, but only rats trained in the tasks demanding hippocampal participation presented changes in theta expression. Moreover, the changes observed in the RP in the CTR group in this study were prominent on days 5 and 6 of training, whereas changes associated with familiarity with the environment must be evident on the first days of training. Although learning could be divided into stages (essentially for the purposes of the study), learning and the consolidation of learning could occur throughout the entire training of the rats in long-term paradigms, such as the water maze. In this paradigm, there is no clear threshold indicating when the animal is learning and when it is recovering information learned in previous trials or on previous days of training; although the acquisition of more precise information continues occurring and allows the animal to develop over the days direct pathways toward the platform, it is logical to suppose that, over the training days, both consolidation of some information (spatial, motor, proprioceptive) acquired in the first trials or days could occur, whereas other information is acquired. Moreover, the recovery of the previously acquired information could occur from trial to trial or day to day of training. Thus, the different weights of the place learning processes presumably occurred at different times; it is reasonable to assume that higher acquisition of information occurred during the first 2 days, and higher consolidation and recovery of information occurred on the last 2 days, supporting the view that the processes occurred simultaneously. As in the previous work, in which depletion of SUMn/PH was realized (Gutiérrez-Guzman et al., 2012), a lack of learning-related changes in theta activity for RP was observed during processing of spatial information, not only in CA1 but also in all of the regions recorded.

The SUMn serotonin-depleted rats failed to show the increase in high-frequency theta RP related to changes over time during training, this was evident both in the peak frequency and in the RP. This failure could imply that serotonin participates in the SUMn-driven regulation of hippocampal frequency. In anesthetized rats, it was observed that neuronal SUMn theta-related firing predicted the changes in theta activity in hippocampus when sensorial stimulation occurs and also in brief episodes of theta when acceleration in frequency occur; however, the hippocampus drives the SUMn activity during spontaneous theta trains (Kocsis and Kaminski, 2006). Additionally, it has been previously observed that the ascending influence of the SUMn on hippocampal theta is not required for the occurrence theta, but it was proposed that the SUMn coding of theta frequency becomes relevant when there is a high degree of processing of information (Kirk and Mackay, 2003). Based on the absence of differences in AP through the days, we can hypothesize that changes in RP associated to learning may be caused by the same population of neurons tuning their synaptic oscillations within the range of the theta band, from lower to higher frequencies, effect that was absent in the EXP group. Thus, an increase in RP of one hertz (e.g., 8 Hz) could occur when, in fact, their power increased or when the power of all of the other frequencies decreased, or the two phenomena occurred simultaneously whatever the mechanism, it implies the predominance of high frequency activity.

The changes in RP coherence in CTR rats could reflect increased communication between the MS and hippocampus possibly related to consolidation of spatial information and recovery of the same. In accordance, it was reported that hippocampus weakly conduced SUMn activity during the initial training, in a 1-day test of spatial learning (16 trials), whereas during the last trials of training the direction of the influence inverted so that the SUMn directed the hippocampal activity, which was also associated with an increase in coherence between the two regions during the last training trials, when the consolidation of information takes place (Ruan et al., 2011). Differences in the training paradigm could account for this because in the present work, 4 daily trials were given to the rats and more gradual process of consolidation could be occurring in comparison with the collapsed training (16 trials) in 1 day. However, in the present work, we did not observe increased coherence between the SUMn and hippocampus across training days in control rats, but the frequency of the peak of coherence for DG-SUMn increased with the days, to be significant on day 6; moreover increased coherence was evident between the MS and the hippocampus (DG and CA1) on days in which consolidation occurred more preferentially (days 3–6); and increase in the frequency of the coherency peak occurred for MS-DG (gradual but significant on day 6).

**Figure 10:**
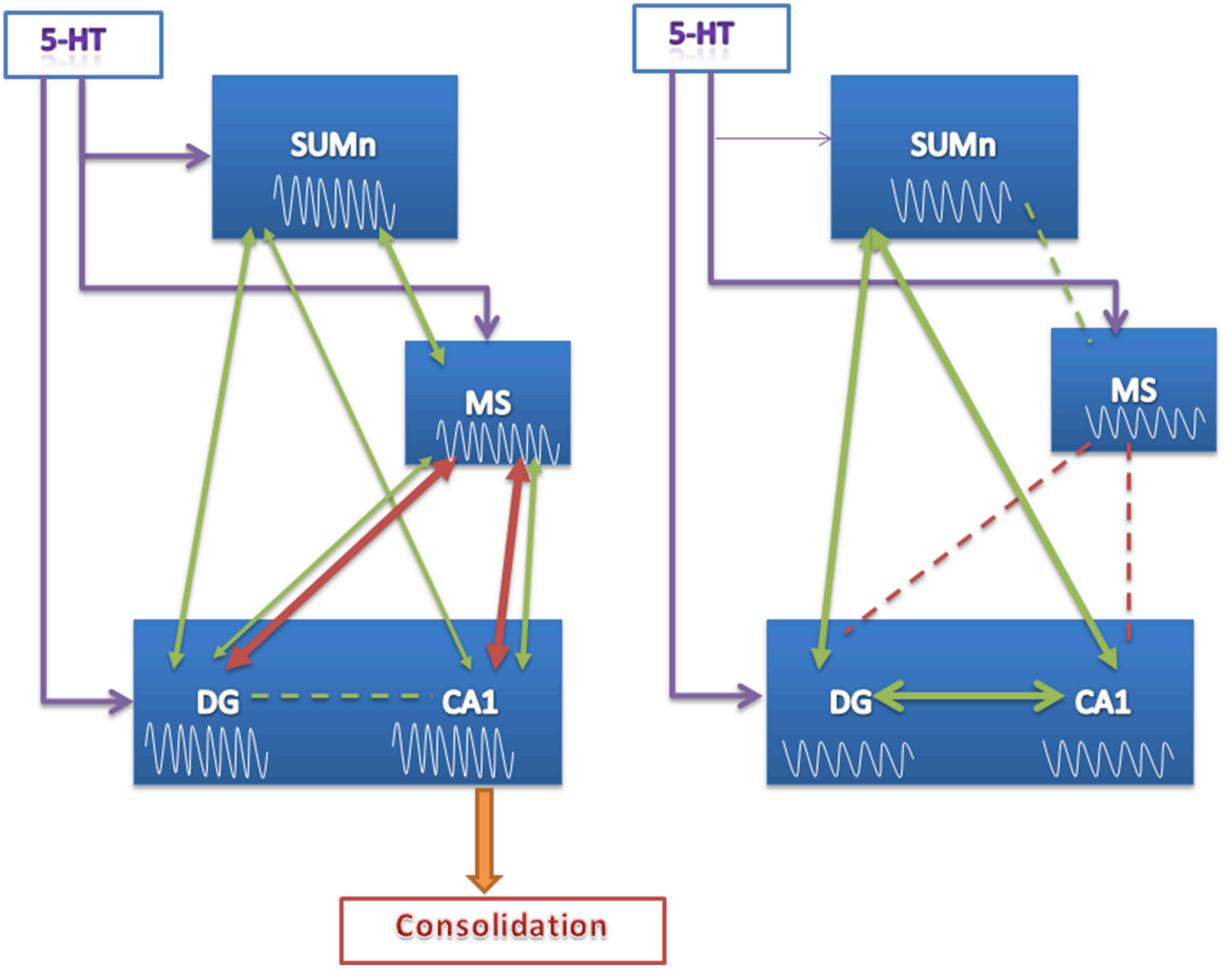
**Proposed model of function of the SUMn, the MS and the hippocampus circuit during learning after SUMn serotonin-depletion**. The model represents the results observed principally the last days of training. EEG RP values are represented into the boxes, showing that no increase in high frequency RP was observed in the EXP group. Green arrows represent values of frequency peak; red arrows represent values of coherence. The thickness of the arrows represent the grade of coherence or correlation; broken lines represent the lack of coherence or correlation. When 5-HT was reduced in the SUMn, the circuit appear to entrain into a closed mode between SUMn and hippocampus. The lack of communication between MS and CA1 would impair the consolidation of memory.

Thus, the learning of the spatial task was accompanied by changes in power in all regions recorded and increased coherence between MS and the hippocampus across training days in CTR animals; these changes were absent in the EXP group. Surprisingly, MS theta activity did not show changes in relation to the SUMn in coherence, and the RP increased only in the 8 Hz frequency across training days, this was unexpected in view of the modulator role of the SUMn on MS activity. The absence of increases in the RP of high-frequency theta in the SUMn serotonin-depleted rats as well as the absence of increases in coherence between the MS and the hippocampus could underlie the inefficient performance of these animals. In support of this idea, the peak frequency showed in each region was highly correlated in CTR animals (even though scant direct connection has been reported between the SUMn and the CA1) (Haglund et al., 1984), whereas in the EXP group the RP peak frequency between MS and the other two regions was unrelated, and there were highly correlated peak frequencies within the hippocampus (DG with CA1) and between hippocampus and the SUMn. This result, together with the minor coherence between the MS and the CA1 and the MS and the DG in SUMn serotonin-depleted animals (compared with the CTR group) would imply a reduced communication between the MS and the hippocampus caused by the withdrawal of the SUMn serotonin influence. The influence of SUMn could be necessary to entrain the information flux in the MS-hippocampus circuit, during consolidation of memory, and the absence of serotonin appears to alter the fine-tuning of the SUMn activity required for this purpose. Instead, the EXP animals appear to be entrained in a closed circuit between the hippocampus and SUMn, and this would impair the consolidation of memory (Figure 10).

The SUMn receives projections from medial and lateral mammillary (MM) nuclei (Gonzalo-Ruiz et al., 1992), and the MM theta-related rhythmic firing originates from a descendent influence from the hippocampus, whereas SUMn theta influences ascend through the input received from the RPOn and TPPn (Kocsis and Vertes, 1994; Kirk et al., 1996; Kirk, 1997). Although in the present work we cannot rule out the possibility of some leakage of 5,7-DHT to the adjacent MM region, the descendent origin of MM theta supports the idea that the changes observed in the EXP group are mainly due to SUMn serotonin depletion. Moreover, the role of the MM in place learning has been evaluated, and no changes in a spatial reference memory similar to those reported in the present work were observed, although, mild to severe deficiencies occurred after total or partial MM lesions when spatial working memory was implicated, e.g., in T maze delayed tasks and in Morris maze and radial arm maze working memory tasks (Santin et al., 1999; Vann and Aggleton, 2003; Vann, 2005). A previous study reporting place reference memory deficits after MM lesions included animals that suffered bilateral destruction of the SUMn in addition of the MM damage (Sutherland and Rodriguez, 1989); moreover, increased cFos expression occurred in the medial MM nucleus after a working memory task but not after a spatial reference memory task (Santin et al., 2003). Thus, it is unlikely that the deficiencies observed in spatial reference memory in Morris maze in the present work were due to serotonin depletion that extended into the MM.

The SUMn is monosynaptically connected to the DG in a segregated pattern, the lateral SUMn synapses with the dorsal DG and the medial SUMn synapses with the ventral DG (Ohara et al., 2013), where it makes glutamatergic and GABAergic/Glutamatergic contacts both on granule cells and on GABAergic neurons (Nitsch and Leranth, 1996). The SUMn also sends glutamatergic afferents to the CA2/CA3 regions of the hippocampus (Soussi et al., 2010), and is also reciprocally connected to the MS (Vertes, 1988, 1992), through a glutamatergic input onto cholinergic and GABAergic MS neurons and a GABAergic descending input from the lateral septum (LS) onto the lateral SUMn (Leranth and Kiss, 1996). Unlike the abundance of knowledge about the connectivity of the SUMn, there is scant information about the serotonergic projections to and receptors, through which serotonin influences the neuronal activity, on the SUMn. A moderate concentration of serotonergic terminals was reported to project to the lateral SUMn and slightly denser concentration was reported in the medial SUMn (Moore et al., 1978; Vertes and Martin, 1988; Vertes et al., 1999). In addition, the presence of 5HT_1C_ and 5-HT_2_ receptors, particularly the 5-HT2A receptor with a moderate density both on the soma and on dendrites of neurons, has been reported on the SUMn (Wright et al., 1995; Cornea-Hébert et al., 1999). Whereas the effect of SUMn manipulations on CA2/CA3 is relatively unexplored, CA1 pyramidal excitability is suppressed and theta activity activated by SUMn carbacol microinjections (Jiang and Khanna, 2006) and SUMn stimulation increases the population of spikes in the DG evoked by stimulation of the perforant pathway in anesthetized rats (Mizumori et al., 1989). Additionally, the SUMn is known to modulate septal cell firing and hippocampal theta frequency in anesthetized rats, in which procaine injection into SUMn produced the attenuation of both frequency and amplitude of hippocampal theta (Kirk and McNaughton, 1993); however, it was observed that the electrolytic lesioning of the SUMn did not affect the movement-related theta frequency in behaving rats (Thinschmidt et al., 1995). In this manner, it is highly speculative attempt to explain what could be the consequence of reduced serotonin on the electrical activity at the neuronal level in the SUMn and the repercussion on the MS. Although it is possible support that the tuning of theta during movement-related information processing (e.g., place learning) and not the movement-related theta could be disrupted in SUMn serotonin-depleted rats, this remains speculative. To our knowledge, no other evidence of SUMn modulation of theta activity during learning in awake rats exists; however, whatever the effect, the present results support the role of the serotonin acting on the SUMn, in the modulation of the hippocampal theta activity underlying the processing of spatial information and in the consolidation of this information.

In conclusion, reduction of serotonin in the SUMn produced deficiencies in place learning ability and altered pattern of hippocampal, septal, and SUMn theta learning-related activity, in the rat.

## Author contributions

All authors participated in the experimental design, experimental work and data analysis. In addition, JH and MO participated in the redaction of the final article and all four authors discussed the contents and interpretations of the work.

### Conflict of interest statement

The authors declare that the research was conducted in the absence of any commercial or financial relationships that could be construed as a potential conflict of interest.
