# Supplementary material for: Supramammillary serotonin reduction alters place learning and concomitant hippocampal, septal, and supramammillar theta activity in a Morris water maze
Source: Front Pharmacol. 2015 Oct 29;6:250. doi: 10.3389/fphar.2015.00250 (PMC4625187; doi:10.3389/fphar.2015.00250)
Supplement: Supplementary file 1 [file DataSheet1.DOCX]

Supplementary results

In order to know if a shift occurred through the training days in the frequency in which the peak of coherence occurred (frequency of the coherence peak, FCP), FCP was compared between days of training in both groups of animals. In the CTR group MS-DG FCP did not show increased frequency when compared the six days of training, but was significant when compared only the days 1, 2, 5 and 6 [F (3, 27) = 3.451, p = 0.038]. Paired comparisons showed increased FC) on day 6 compared with the first day of training, whereas the magnitude of the coherency peak [F (5, 41) = 6.22, p = 0.0005] was higher on days 4, 5 and 6 compared with the first day (Figure S1). MS-CA1 FCP also showed changes when compared days 1, 2 5 and 6 [F (3, 27) = 3.356, p = 0.041], the FCP was higher the day 6 compared with the day 2; the magnitude of the peak of coherence significantly increased [F (5, 41) = 11.28, p <0.0001], and was higher on days four to six, compared with the day one. MS-SUM FCP did not show changes through the days, but the magnitude of the coherence peak significantly increased [F (5, 1) = 8.760, p < 0.0001] on days 3 to 6, compared with the day 1. DG-SUM FCP significantly changed with the training days, only comparing the days 1, 2, 5 and 6 [F (3, 27) = 3.044, p = 0.055], paired comparisons showed on day 6 higher FCP compared with the first day. Additionally, the magnitude of the coherence peak [F (5, 41) = 4.408, p = 0.004] increased on day five compared with the days 1 and 2. CA1-SUM FCP did not show changes through the training days, but showed the increased magnitude of the coherence peak [F (5, 41) = 5.707, p = 0.0008], on day 5 compared with the days 1 and 2. Finally, the DG-CA1 FCP did not show significant changes through the days, but the magnitude of the coherence peak increased significantly [F (5, 41) = 0.0008] on days 5 and 6 compared with the days 1 and 2. The EXP group did not show changes in the FCP through the training days in any of the pairs of regions. Whereas, in the magnitude of the coherence peak, the EXP group sowed changes in MS-CA1 [F (5, 35) = 4.373, p = 0.0054], on day 6 compared with the day 1 and 2; in MS-SUM [F (5, 35) = 6.689, p = 0.0004], on days 5 and 6 compared with the first day; and finally in the DG-SUM [F (5,35) = 3.171, p = 0.0237], on day six compared with the first day. Inter-group comparisons of FCP were significant for MS-CA1 (Main effect) [F (1, 60) = 20.39, p = 0.0058]; and CA1-SUM (main effect) [F (1, 60) = 11.87, p = 0.048], in both cases the FCP were lower for the EXP group. Additionally, the magnitude of the peak of coherence was significantly different from MS-CA1 (main effect) [F (1, 60) = 8.633, p = 0.124], the EXP group had a lower peak coherence magnitude, and also a significant interaction of the group and day was observed [F (5, 60) = 2.858, p = 0.0222], paired comparisons showed lower magnitude of the coherence peak magnitude on days 3 and 5 for the EXP group compared with the CTR group (Figure S1).

Figure S1. The frequency of the coherency peak (FCP) (left y axe), and magnitude of the peak of coherence (right y axe), of the groups CTR (black circles) and EXP (red squares), for the different pairs of regions recorded through the training days. Values are mean ± SEM. *, day 1 vs. subsequent days; **°**, day 2 vs. subsequent days; for the group CTR (black) or EXP (red). +, CTR group vs. EXP group.
