# Supplementary figures and images for: Supramammillary serotonin reduction alters place learning and concomitant hippocampal, septal, and supramammillar theta activity in a Morris water maze
Source: Front Pharmacol. 2015 Oct 29;6:250. doi: 10.3389/fphar.2015.00250 (PMC4625187; doi:10.3389/fphar.2015.00250)

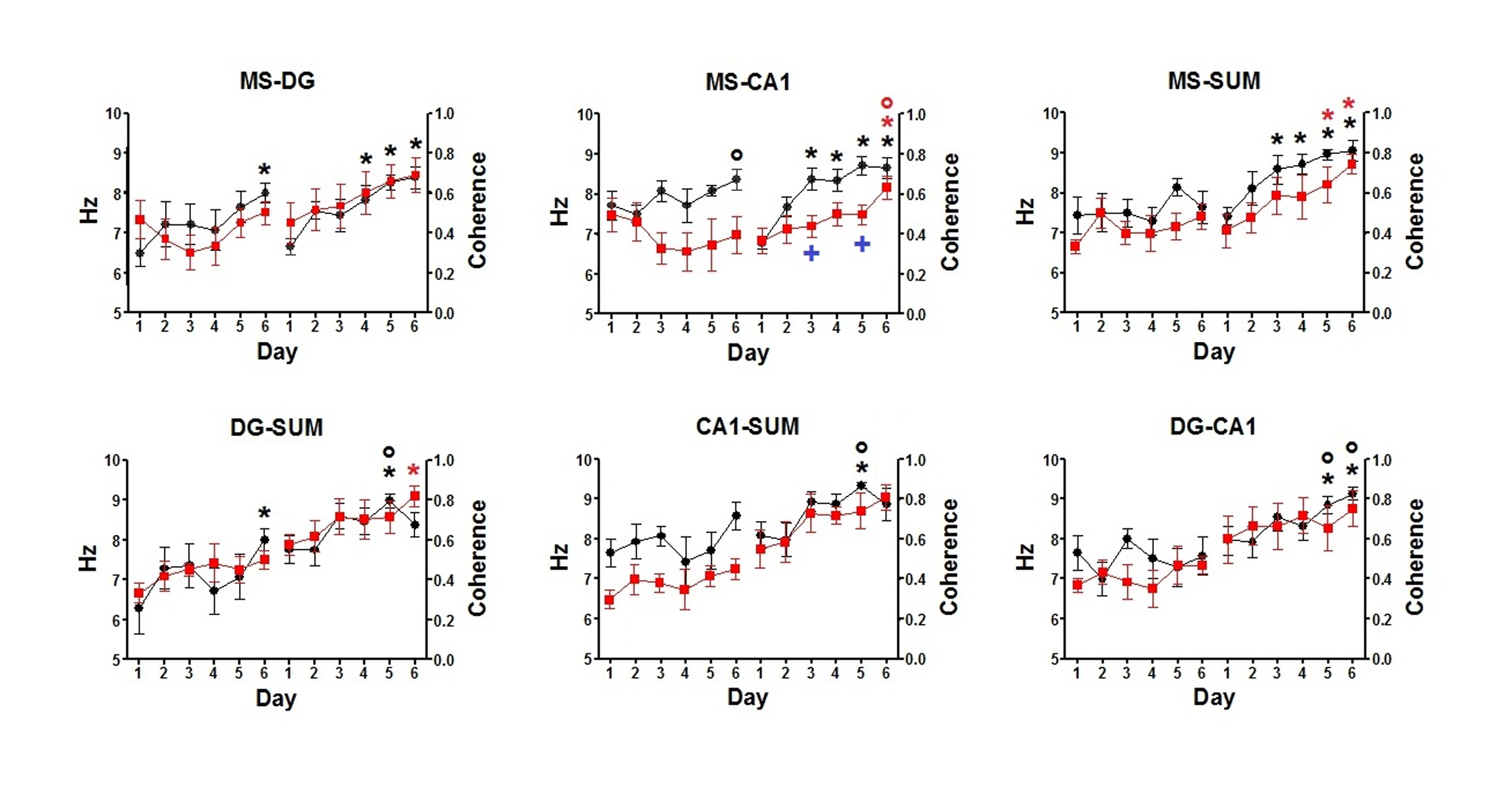

Supplement: Supplementary file 2 [file Image1.TIF]
